# Verification of the methodology for evaluating tumor-infiltrating lymphocytes in colorectal cancer

**DOI:** 10.18632/oncotarget.24612

**Published:** 2018-03-08

**Authors:** Shinji Matsutani, Masatsune Shibutani, Kiyoshi Maeda, Hisashi Nagahara, Tatsunari Fukuoka, Yasuhito Iseki, Kosei Hirakawa, Masaichi Ohira

**Affiliations:** ^1^ Department of Surgical Oncology, Osaka City University Graduate School of Medicine, Osaka, Japan

**Keywords:** tumor-infiltrating lymphocytes, TILs, prognostic marker, colorectal cancer, methodology

## Abstract

**Background:**

The density of tumor-infiltrating lymphocytes (TILs) have been reported to reflect antitumor immune response and correlate with prognosis in malignancy. However, the methodology for evaluating the density of TILs by an immunohistochemical analysis differs among reports. The aim of this study was to verify the methodology for evaluating the density of TILs by immunohistochemical analysis and thereby identify the optimum methodology in clinical setting.

**Methods:**

Three-hundred-thirteen patients who underwent curative operation for stage II/III colorectal cancer were enrolled. We retrospectively examined the density of TILs using immunohistochemical staining according to each method as follows: 1) subset of lymphocytes (i.e. CD4^+^/CD8^+^), 2) selected fields (i.e. at random or focusing on hot spots), 3) location in low-power field (i.e. the invasive margin [TILs^IM^] or the center of the tumor [TILs^CT^] or the surface of the tumor [TILs^ST^]), and 4) location in high-power field (i.e. in tumor stroma [sTILs] or intra-tumor cells [iTILs] or total TILs [tTILs: sTILs+iTILs]). We then assessed the prognostic value of the density of TILs^IM^ evaluated as described above. We also evaluated the correlation between the density of TILs^IM^ and that of TILs^CT^/TILs^ST^.

**Results:**

Only the densities of CD8^+^sTILs^IM^ and CD8^+^tTILs^IM^ evaluated in randomly selected fields were significantly associated with the survival. Furthermore, the density of CD8^+^TILs^IM^ was significantly associated with that of CD8^+^TILs^CT^ and CD8^+^TILs^ST^.

**Conclusions:**

We concluded that best and easiest way to evaluate the density of TILs in the clinical setting may be to assess the density of CD8^+^tTILs^IM^ in randomly selected fields.

## INTRODUCTION

Colorectal cancer (CRC) is the third-most common cancer worldwide, with a cumulative lifetime risk of approximately 5% [1, 2], and the clinical outcome of CRC is poor, as one-third of patients who undergo curative resection die within 5 years after surgery [3]. To identify patients at high risk of disease recurrence, AJCC/UICC-tumor-node-metastasis (TNM) classification is employed most frequently as a prognostic classification. However, the prognostic value of this system is limited [4]. Therefore, genetic and molecular tumor prognostic factors have alternatively been proposed to identify patients who may be at risk for recurrence. However, none of these have been sufficiently informative for inclusion in clinical practice [5]. The identification of patients at high risk of disease recurrence therefore remains a major clinical issue.

As the primary host immune response against malignant tumors, tumor-infiltrating lymphocytes (TILs) have been reported to have a crucial effect on tumor progression and the clinical outcome in various types of cancer, including non-small cell lung cancer (NSCLC), colorectal, esophageal, and urothelial cancers and melanoma [6–13]. Furthermore, Galon et al. [14] reported that the density of TILs are more valuable prognostic markers than the TNM classification. However, while a number of methods have been proposed for evaluating the density of TILs, none has yet been confirmed to be optimum.

Some researchers have evaluated the density of TILs in Hematoxylin-Eosin-stained sections, and others have evaluated the density of the subset of TILs in immunohistochemical-stained sections. The methodology for evaluating the density of TILs by immunohistochemical staining differs among reports, with suggested methods as follows: selected fields (i.e. at random or focusing on hot spots), location in high-power field (i.e. in tumor stroma, intra-tumor cells, and total TILs), and location in low-power field (i.e. the invasive margin, the center of the tumor, surface of the tumor). As described above, no standard methodology for evaluating the density of TILs has yet been established. Therefore, a standardized methodology for evaluating the density of TILs is required in order to apply this biomarker in the clinical setting.

The aim of this study was to identify the optimum methodology for evaluating the density of TILs by immunohistochemical staining to help predict the prognosis of patients.

## RESULTS

### Patients’ characteristics in the exploratory study

The patient characteristics are listed in Table 1. The resected specimens were pathologically classified according to the seventh edition of the UICC TNM classification of malignant tumors. The distribution of cancer stages was as follows: stage II, 72; stage III, 67 patients. Mismatch repair status was as follows: proficient, 133; deficient, seven patients. All patients were followed up regularly with physical and blood examinations, including measurements of the levels of tumor markers, such as carcinoembryonic antigen (CEA) and carbohydrate antigen 19-9 (CA19-9), and mandatory screening using colonoscopy and computed tomography until August 2016 or death. The median follow-up period for the survivors in this study was 64.0 months (range: 6-107). Seventeen patients died during the follow-up period due to CRC.

**Table 1:** Patients characteristics

	Exploratory Group (n= 139)	Validation Group (n= 174)
Gender		
Male	72	89
Female	67	85
Age (years)		
Median (range)	65 (29-89)	69 (21-96)
Location of primary tumor		
Colon	70	102
Rectum	69	72
Tumor depth^*^		
T1-3	92	117
T4	47	57
Tumor diameter (cm)		
Median (range)	5.0 (1.0-11.0)	4.0 (0.8-12.0)
Histological type		
Well, Moderately	127	162
Poorly, Mucinous	12	12
Lymphatic involvement		
Negative	30	54
Positive	109	120
Venous involvement		
Negative	113	134
Positive	26	40
Lymph node metastases		
Negative	72	96
Positive	67	78
Mismatch repair status		
Proficient	133	164
Deficient	6	10

^*^: According to UICC.TNM Classification of Malignant Tumors (seventh edition).

### A survival analysis for TILs^IM^ in randomly selected field in the exploratory study

**Figure 1:**
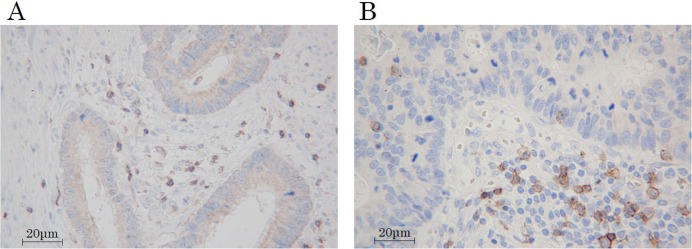
Kaplan-Meier survival curves for the disease-specific survival according to the density of CD4/CD8^+^ TILs at the invasive margin (TILs^IM^) in randomly selected fields in the exploratory study **(A)** CD4^+^ tTILs^IM^. **(B)** CD4^+^ sTILs^IM^. **(C)** CD4^+^ iTILs^IM^. **(D)** CD8^+^ tTILs^IM^. **(E)** CD8^+^ sTILs^IM^. **(F)** CD8^+^ iTILs^IM^. tTILs, total TILs. sTILs, TILs in tumor stroma. iTILs, TILs intra-tumor cells.

We assessed the prognostic value of the density of TILs at the invasive margin, in which the characteristics of the tumor have been recognized to be most accurately reflected [15]. The densities of CD4^+^ tTILs^IM^, sTILs^IM^, and iTILs^IM^ showed no prognostic significance (Figure 1A, 1B, 1C). However, high-CD8^+^ tTILs^IM^ and sTILs^IM^ were significantly associated with high disease-specific survival (DSS) rates (p=0.037, p=0.030, respectively) (Figure 1D, 1E), although the density of CD8^+^iTILs^IM^ was not associated with the prognosis (Figure 1F).

### A survival analysis for TILs^IM^ in hot spots in the exploratory study

**Figure 2:**
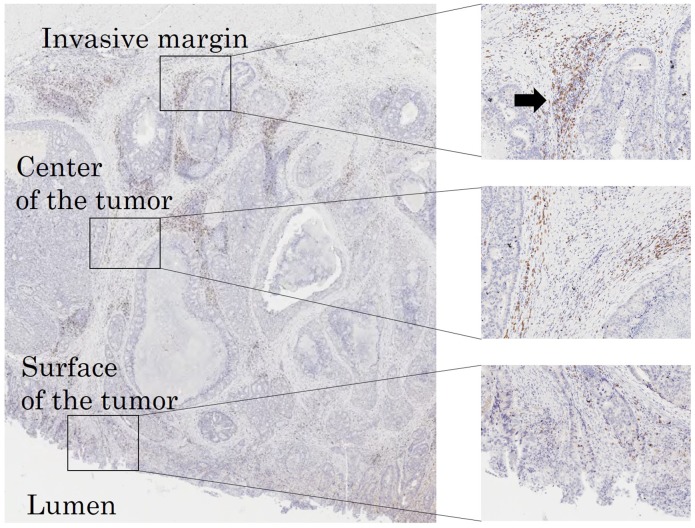
Kaplan-Meier survival curves for the disease-specific survival according to the density of CD4/CD8^+^ TILs at the invasive margin (TILs^IM^) focusing on hot spots in the exploratory study **(A)** CD4^+^tTILs^IM^. **(B)** CD4^+^sTILs^IM^. **(C)** CD4^+^iTILs^IM^. **(D)** CD8^+^tTILs^IM^. **(E)** CD8^+^sTILs^IM^. **(F)** CD8^+^iTILs^IM^. tTILs, total TILs. sTILs, TILs in tumor stroma. iTILs, TILs intra-tumor cells.

In our evaluation focusing on hot spots, the densities of all TILs evaluated by each method showed no prognostic significance (Figure 2).

### Correlations between the density of CD8^+^tTILs^IM^ and the clinicopathological factors in the exploratory study

The density of CD8^+^tTILs^IM^ in randomly selected field exhibited no significant relationship with any of the clinicopathological parameters, except for lymph node metastasis (p=0.028) (Table 2).

**Table 2:** Correlations between the density of CD8^+^tTILs^IM^ and the clinicopathological factors

	Exploratory Group CD8+tTILs^IM^			Validation Group CD8+tTILs^IM^		
	Low	High	p-value	Low	High	p-value
Gender						
Male	33	39		23	66	
Female	36	31	0.398	13	72	0.095
Age (years)						
<65	35	30		14	47	
≥65	34	40	0.397	22	91	0.695
Tumor depth^*^						
T1-3	41	51		26	91	
T4	28	19	0.109	10	47	0.553
Tumor diameter (cm)						
<5	33	32		21	80	
≥5	36	38	0.866	15	58	1.000
Tumor location						
Right side	19	14		11	47	
Left side	50	56	0.325	25	91	0.843
Histological type						
Well, Moderate	64	63		32	130	
Poorly, Mucinous	5	7	0.764	4	8	0.274
Lymphatic involvement						
Negative	12	18		9	45	
Positive	57	52	0.303	27	93	0.425
Venous involvement						
Negative	53	60		25	109	
Positive	16	10	0.198	11	29	0.267
Lymph node metastasis						
Negative	29	43		19	77	
Positive	40	27	0.028	17	61	0.851
CEA (ng/ml)						
<5	39	48		22	80	
≥5	30	22	0.163	14	56	0.851
CA19-9 (U/ml)						
<37	58	65		32	123	
≥37	8	5	0.389	3	12	1.000

^*^: According to UICC.TNM Classification of Malignant Tumors (seventh edition).

CEA, carcinoembryonic antigen; CA19-9, carbohydrate antigen 19-9.

### Correlations between the MMR status and the density of CD8^+^tTILs^IM^ in the exploratory study

**Figure 3:**
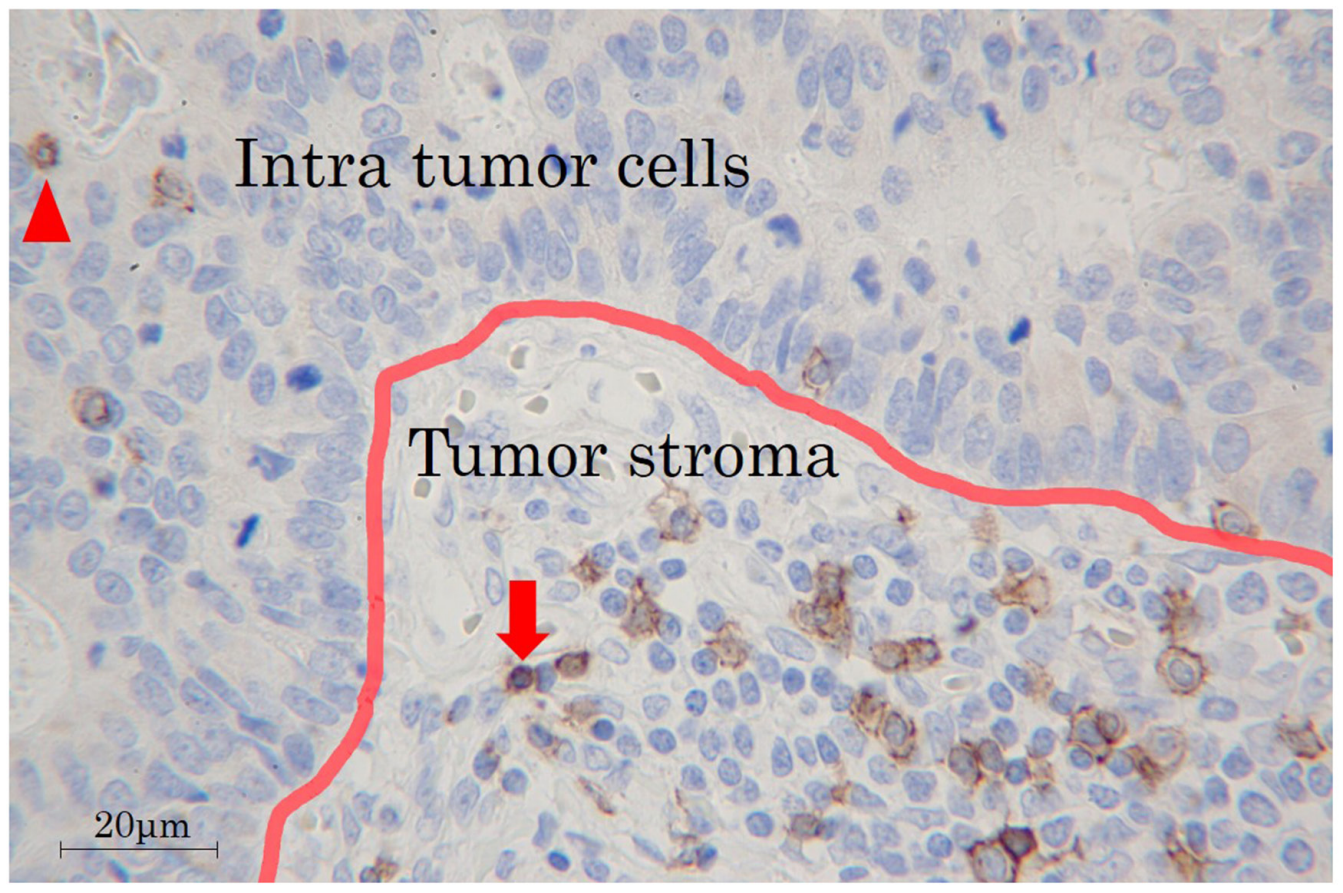
The correlations between mismatch repair status and the density of total CD8^+^TILs at the invasive margin (CD8^+^tTILs^IM^) in randomly selected fields ^*^p < 0.1, ^**^p < 0.05.

The density of CD8^+^tTILs^IM^ in randomly selected fields in MMR-D patients tended to be higher than that in MMR-P patients (p=0.077) (Figure 3A).

### Prognostic factors influencing the survival in the exploratory study

The correlations between the DSS and various clinicopathological factors are shown in Table 3. A multivariate analysis indicated that none of the factors were independent prognostic factors for the DSS.

**Table 3:** Correlations between the disease-specific survival and various clinicopathological factors in the exploratory study

	Univariate analysis			Multivariate analysis		
	HR	95%CI	p-value	HR	95%CI	p-value
Age (≥65 vs. <65)	2.078	0.754-6.602	0.161	1.485	0.498-4.926	0.482
Tumor depth (T4 vs. T1-3)	2.067	0.760-5.625	0.151	0.920	0.283-3.001	0.889
Histological type (Poorly, Mucinous vs. Well, Moderately)	2.327	0.533-7.228	0.229	1.283	0.226-6.049	0.764
Lymphatic involvement (Positive vs. Negative)	4.522	0.915-81.764	0.068	1.589	0.264-30.523	0.656
Venous involvement (Positive vs. Negative)	3.042	1.034-8.206	0.044	2.550	0.795-7.630	0.111
Lymph node metastasis (Positive vs. Negative)	5.244	1.705-22.791	0.003	2.247	0.625-10.771	0.225
CEA (≥5 ng/ml vs. <5 ng/ml)	5.905	2.086-20.987	<0.001	2.922	0.881-11.308	0.080
CA19-9 (≥37 U/ml vs. <37 U/ml)	6.348	1.968-17.975	0.004	2.730	0.598-10.723	0.184
The density of CD8^+^tTILs^IM^ in randomly selected field (Low vs. High)	3.135	1.092-11.216	0.033	1.886	0.567-7.424	0.308
Mismatch repair status (deficient vs. proficient)	<0.001	NA	0.223	<0.001	0.000-33.366	<0.999

HR, hazard ratio; CI, confidence interval; CEA, carcinoembryonic antigen; CA19-9, carbohydrate antigen 19-9; tTILs^IM^, total tumor-infiltrating lymphocytes at the invasive margin; NA, not available.

### Correlation between the density of TILs^IM^ and TILs^CT^/TILs^ST^ in the exploratory study

**Figure 4:**
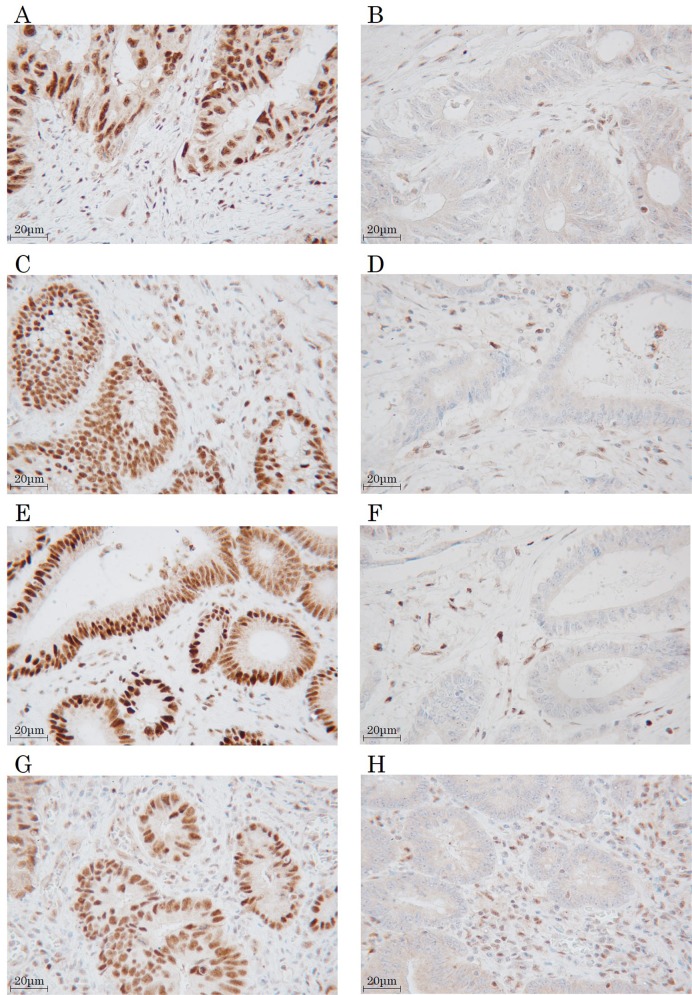
The correlations between the densities of total CD8^+^TILs at the center of the tumor (CD8^+^tTILs^CT^)/at the surface of the tumor (CD8^+^tTILs^ST^) and that of total CD8^+^TILs at the invasive margin (CD8^+^tTILs^IM^) in randomly selected fields in the exploratory study **(A)** center: r=0.705, p<0.001. **(B)** surface: r=0.568, p<0.001.

The densities of CD8^+^tTILs^CT^ and CD8^+^tTILs^ST^ were significantly associated with that of CD8^+^tTILs^IM^ in randomly selected fields (TILs^CT^ (Figure 4A): r=0.71, p<0.001; TILs^ST^ (Figure 4B): r=0.57, p<0.001).

### Patients’ characteristics in the validation study

The patient characteristics are listed in Table 1. The distribution of cancer stages was as follows: stage II, 96 patients; stage III, 78 patients. All patients were followed up as described above until September 2017 or death. The median follow-up period for the survivors in this study was 62.1 months (range: 13-89). Eighteen patients died during the follow-up period due to CRC.

### A survival analysis for TILs^IM^ in randomly selected fields in the validation study

**Figure 5:**
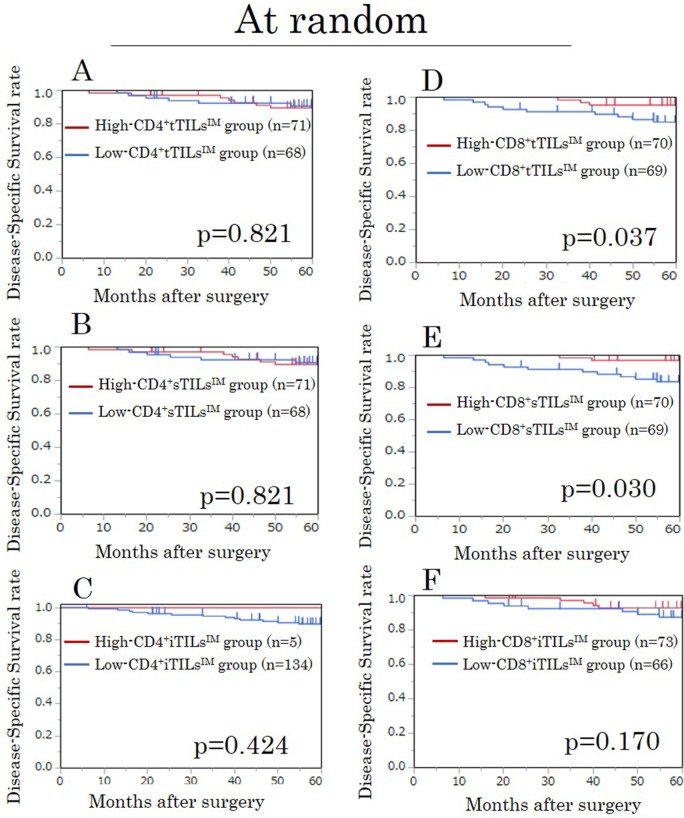
Kaplan-Meier survival curves for the disease-specific survival according to the density of CD4/CD8^+^ TILs at the invasive margin (TILs^IM^) in randomly selected fields in the validation study **(A)** CD4^+^ tTILs^IM^. **(B)** CD4^+^ sTILs^IM^. **(C)** CD4^+^ iTILs^IM^. **(D)** CD8^+^ tTILs^IM^. **(E)** CD8^+^ sTILs^IM^. **(F)** CD8^+^ iTILs^IM^. tTILs, total TILs. sTILs, TILs in tumor stroma. iTILs, TILs intra-tumor cells.

We assessed the prognostic value of the density of TILs at the invasive margin. High-CD8^+^tTILs^IM^/sTILs^IM^ were significantly associated with high DSS rates, just as in the exploratory study (both p<0.001) (Figure 5D, 5E). In addition, high-CD8^+^iTILs^IM^ tended to be associated with high DSS rates (p=0.069) (Figure 5F).

### A survival analysis for TILs^IM^ in hot spots in the validation study

**Figure 6:**
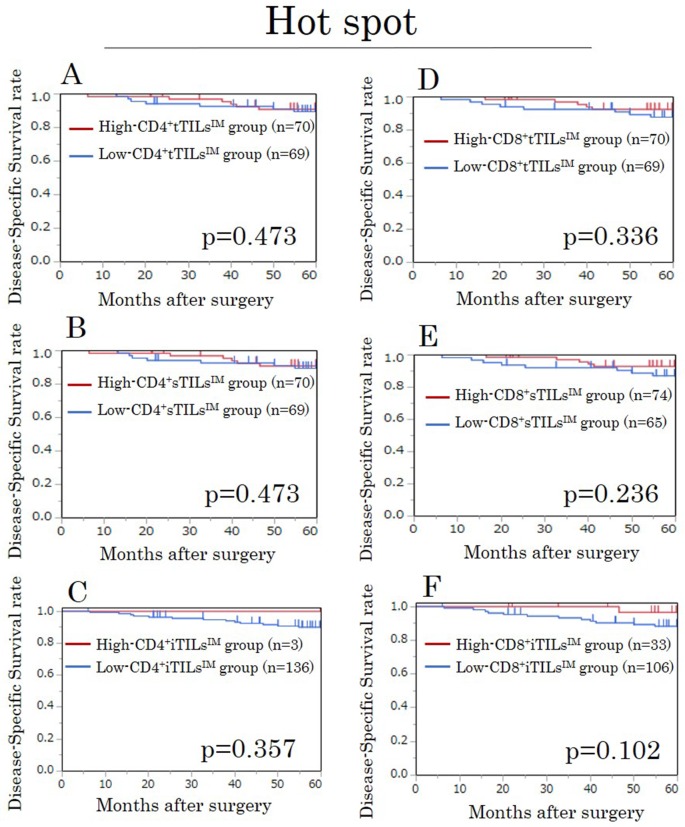
Kaplan-Meier survival curves for the disease-specific survival according to the density of CD4/CD8^+^ TILs at the invasive margin (TILs^IM^) focusing on hot spots in the validation study **(A)** CD4^+^tTILs^IM^. **(B)** CD4^+^sTILs^IM^. **(C)** CD4^+^iTILs^IM^. **(D)** CD8^+^tTILs^IM^. **(E)** CD8^+^sTILs^IM^. **(F)** CD8^+^iTILs^IM^. tTILs, total TILs. sTILs, TILs in tumor stroma. iTILs, TILs intra-tumor cells.

In the evaluation focusing on hot spots, the densities of all TILs evaluated by each method showed no prognostic significance, just as in the exploratory study (Figure 6).

### Correlations between the density of CD8^+^tTILs^IM^ and the clinicopathological factors in the validation study

The density of CD8^+^tTILs^IM^ in randomly selected fields exhibited no significant relationship with any of the clinicopathological parameters (Table 2).

### Correlations between the MMR status and the density of CD8^+^tTILs^IM^ in the validation study

The density of CD8^+^tTILs^IM^ in randomly selected fields in MMR-D patients was significantly higher than that in MMR-P patients (p=0.012) (Figure 3B).

### Prognostic factors influencing the survival in the validation study

The correlations between the DSS and various clinicopathological factors are shown in Table 4. A multivariate analysis indicated that lymph node metastasis (hazard ratio, 4.30; 95% confidence interval, 1.26-20.03; p=0.019) and the density of CD8^+^tTILs^IM^ in randomly selected fields (hazard ratio, 14.94; 95% confidence interval, 4.65-60.02; p<0.001) was an independent prognostic factor for the DSS.

**Table 4:** Correlations between the disease-specific survival and various clinicopathological factors in the validation study

	Univariate analysis			Multivariate analysis		
	HR	95%CI	p-value	HR	95%CI	p-value
Age (≥65 vs. <65)	0.916	0.361-2.491	0.857	0.895	0.293-2.814	0.845
Tumor depth (T4 vs. T1-3)	1.813	0.692-4.599	0.218	1.603	0.390-6.530	0.505
Histological type (Poorly, Mucinous vs. Well, Moderately)	6.537	1.845-18.337	0.006	1.557	0.384-5.528	0.513
Lymphatic involvement (Positive vs. Negative)	8.388	1.723-151.072	0.004	3.765	0.652-71.445	0.155
Venous involvement (Positive vs. Negative)	1.872	0.652-4.823	0.229	0.749	0.190-2.638	0.660
Lymph node metastasis (Positive vs. Negative)	7.053	2.326-30.449	<0.001	4.296	1.258-20.029	0.019
CEA (≥5 ng/ml vs. <5 ng/ml)	2.636	1.037-7.167	0.042	2.765	0.955-8.731	0.061
CA19-9 (≥37 U/ml vs. <37 U/ml)	1.523	0.240-5.401	0.596	2.301	0.325-10.372	0.355
The density of CD8^+^tTILs^IM^ in randomly selected field (Low vs. High)	15.918	5.695-56.251	<0.001	14.943	4.647-60.017	<0.001
Mismatch repair status (deficient vs. proficient)	<0.001	NA	0.151	<0.001	0.000-32.813	<0.999

HR, hazard ratio; CI, confidence interval; CEA, carcinoembryonic antigen; CA19-9, carbohydrate antigen 19-9; tTILs^IM^, total tumor-infiltrating lymphocytes at the invasive margin; NA, not available.

### Correlations between the density of TILs^IM^ and TILs^CT^/TILs^ST^ in the validation study

**Figure 7:**
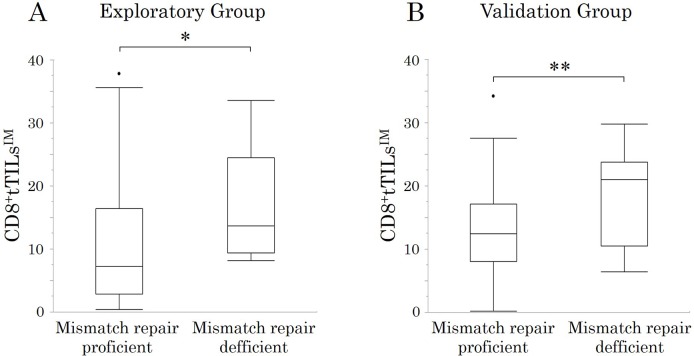
The correlations between the densities of total CD8^+^TILs at the center of the tumor (CD8^+^tTILs^CT^)/at the surface of the tumor (CD8^+^tTILs^ST^) and that of total CD8^+^TILs at the invasive margin (CD8^+^tTILs^IM^) in randomly selected fields in the validation study **(A)** center: r=0.699, p<0.001. **(B)** surface: r=0.610, p<0.001.

The densities of CD8^+^ tTILs^CT^ and CD8^+^ tTILs^ST^ were significantly associated with that of CD8^+^tTILs^IM^ in randomly selected fields, just as in the exploratory study (TILs^CT^ (Figure 7A): r=0.70, p<0.001; TILs^ST^ (Figure 7B): r=0.61, p<0.001).

## DISCUSSION

The current study showed that the densities of both total CD8^+^TILs and CD8^+^TILs in tumor stroma at the invasive margin were associated with the prognosis in patients with Stage II/III CRC. While many previous reports have found the density of TILs as evaluated by immunohistochemical staining to be a useful prognostic marker, the methodology for evaluating the density of TILs has not been standardized. To our knowledge, this is the first report to describe the detailed methodology for evaluating the density of TILs by immunohistochemical staining.

The current study demonstrated that the density of CD4^+^TILs may not be useful as a prognostic marker, while the density of CD8^+^TILs may be useful as a prognostic marker for malignancy. Although some authors have reported that CD4^+^TILs may be a prognostic predictor for malignancies [16, 17], we concluded that the density of CD4^+^TILs was not associated with the prognosis because CD4^+^ T cells can be classified into more detailed subsets, such as T helper 1 (Th1) cells, Th2 cells, Th17 cells, and regulatory T (Treg) cells, and the functions of each CD4^+^ T cell subset differ with regard to antitumor immunity. For example, Th1 cells produce cytokines, such as interferon-γ (INF-γ), which activate CD8^+^ T cells [7]. Therefore, Th1 cells have been reported to enhance the antitumor immune response [18]. However, Th2 cells seem to suppress the antitumor immune response via the activation of B cells or the production of the immunosuppressive cytokine IL-10 [7]. In addition, findings regarding the function of Th17 cells in antitumor immunity have been controversial. For example, some authors have reported that Th17 cells facilitate the antitumor immune response, while other authors have reported that Th17 cells accelerate tumor growth via neoangiogenesis of the tumor [7]. Treg cells have been reported to suppress the antitumor immune response [7]. In contrast, CD8^+^ T cells (cytotoxic T lymphocytes) have been reported to have direct cytotoxic effects on tumor cells via the antitumor immune response and also be strongly associated with prolonged survival [7, 10, 19]. Recently, Galon et al. developed the “immunoscore” as a prognostic indicator using the density of CD8^+^TILs and reported that this score might better reflect the prognosis of cancer patients than the TNM classification [6, 8].

In the current study, the average density of CD8^+^TILs evaluated in five different randomly selected fields was a strong prognostic biomarker. We considered it important to evaluate the antitumor immune status of the whole tumor by evaluating the density of TILs in multiple fields selected randomly in order to resolve the issue of the heterogeneity of the density of TILs in the tumor [20]. However, many previous reports have not described the methodology used to select the fields in which the density of TILs was evaluated [15, 21]. The absence of a consistent methodology for selecting fields may prevent us from accurately evaluating the antitumor immune status. The current study showed that the number of CD8^+^TILs evaluated by focusing on hot spots was not associated with the survival, although a previous report in a large cohort showed that the number of CD8^+^TILs evaluated in areas containing hot spots was significantly associated with the survival [10]. We considered that the number of CD8^+^TILs evaluated in large areas containing hot spots using an image analyzer well-reflected the average density of TILs (i.e. the antitumor immune status in the whole tumor) [10]. In contrast, the number of CD8^+^TILs evaluated by focusing on hot spots may not reflect the antitumor immune status in the whole tumor, because observers evaluated the density of CD8^+^TILs in extremely small areas (i.e. high-power fields) in the current study. We may therefore have incorrectly categorized some patients with low lymphocyte infiltration as having high lymphocyte infiltration. The density of CD8^+^TILs should be evaluated not in the fields focusing on hot spots but in those selected randomly when performing observer-based evaluations.

We found that the density of CD8^+^TILs intra-tumor cells was not a useful prognostic biomarker, despite previous reports on the prognostic utility of the density of CD8^+^TILs intra-tumor cells [22, 23]. The density of CD8^+^TILs intra-tumor cells may be unlikely to reflect differences in the antitumor immune status among patients sensitively, as the absolute number of CD8^+^TILs intra-tumor cells evaluated in high-power fields was quite low. CD8^+^TILs intra-tumor cells, which showed a markedly low density in our study, may not be a useful prognostic biomarker in the clinical setting, although CD8^+^TILs intra-tumor cells may have biological significance.

We found that evaluating the density of total CD8^+^TILs without distinguishing between TILs in tumor stroma and TILs intra-tumor cells was an ideal and easy-to-perform method in the clinical setting. Previous studies in large cohorts [10, 21] have shown that a high density of total CD8^+^TILs was associated with a good survival. Furthermore, the prognostic indicator “immunoscore” [24] described above included the evaluation of the density of total CD8 ^+^TILs. In our results, the number of total TILs was similar to that of TILs in tumor stroma, as the number of TILs intra-tumor cells was extremely low. We therefore considered that the evaluation of the density of total TILs was more reasonable than the evaluation of that of TILs in tumor stroma in analyses performed using an image analyzer [10, 15, 25], which has difficulty distinguishing between TILs intra-tumor cells and TILs in tumor stroma. Furthermore, the evaluation of total TILs without distinguishing between TILs intra-tumor cells and TILs in tumor stroma was a useful and easy-to-perform method for evaluations carried out by an observer.

High-grade MSI (MSI-H) CRC is reportedly more immunogenic with greater infiltration by immune cells than microsatellite stable (MSS) CRC because of the large number of tumor antigens produced by frameshift mutations [26, 27]. Patients with MSI-H Stage II/III CRC have been found to have a better prognosis than those with MSS CRC [28], because MSI-H tumors are suppressed by a strong antitumor immune response associated with MSI-H tumors. Based on these findings, the MSI status may induce a bias in the association between the density of TILs and the prognosis. Although MMR-D tumors had greater CD8^+^TIL infiltration than MMR-P tumors in the current study, relatively few CRC patients had MMR-D tumors (5.1%), and the density of CD8^+^TILs was an MMR status-independent prognostic biomarker.

In previous reports, when assessing the antitumor immunity, most researcher have evaluated the density of TILs at the invasive margin [15, 21] or the combination of the density of TILs at the invasive margin and those of TILs at the center of the tumor [8, 10, 25]. On the other hand, the density of TILs at the surface of the tumor in pretreatment biopsy samples of rectal cancer was recently reported to be useful as a marker for predicting the response to neoadjuvant therapy in patients with locally advanced rectal cancer [29–31]. However, whether or not the density of TILs at the surface of the tumor accurately reflects the antitumor immune status of the whole tumor has been unclear. The current study showed that the density of TILs at the surface of the tumor was significantly associated with that of TILs at the invasive margin. We therefore concluded that the density of TILs at the surface of the tumor may reflect the antitumor immune status to some extent and may be secondary biomarkers of the antitumor immune status. This notion supports the findings of previous reports regarding the utility of assessing the antitumor immune status by evaluating the density of TILs at the surface of the tumor in pretreatment biopsy samples of rectal cancer as a predictive marker for response to neoadjuvant therapy [29–31].

Several limitations associated with the present study warrant mention. First, the current study was retrospective with relatively few patients. Second, we did not evaluate the fine subsets of CD4^+^TILs and CD8^+^TILs. Future studies should investigate the significance of the fine subsets of CD4^+^cells (i.e. Th1, Th2, Th17, Treg cells) and CD8^+^cells (i.e. CD8^+^ memory T cells) in antitumor immunity. Third, the optimum methodology of evaluating the density of TILs has not been established. In the current study, we counted the absolute number of TILs in order to evaluate the antitumor immune status. However, Salgado et al. [32] evaluated the percentage of the area occupied by TILs in the tumor stroma area as a semiquantitative parameter (every 10%), and Richards et al. [23] evaluated the density of TILs semi-quantitatively as absent, weak, moderate, or strong. Applying the evaluation of TILs in the clinical setting will require determining the optimum methodology of measuring the density of TILs. Fourth, in the current study we evaluated the average number of TILs in five different randomly selected fields in the tumor in order to resolve the issue of the heterogeneity of TILs. However, this issue still remains, making it necessary to establish a better method of evaluating the antitumor immune status for the whole tumor.

## CONCLUSIONS

We concluded that the best and easiest way to evaluate the density of TILs in the clinical setting may be to assess the density of total CD8^+^TILs at the invasive margin in randomly selected fields.

## PATIENTS AND METHODS

### Patients

A total of 313 patients with stage II/III CRC were enrolled in this study. All patients underwent potentially curative surgery for CRC at the Department of Surgical Oncology of Osaka City University between 2007 and 2012. Patients who received preoperative therapy, underwent emergency surgery for perforation/obstruction, or who had inflammatory bowel disease were excluded from this study.

All patients were divided into two groups: including the exploratory group, which consisted of 139 patients who underwent surgery between 2007 and 2009; and the validation group, which consisted of 174 patients who underwent surgery between 2010 and 2012.

### Immunohistochemistry for CD4/CD8

Surgically resected specimens were retrieved in order to perform the immunohistochemistry. All 4-μm-thick sections were deparaffined and rehydrated and then subjected to endogenous peroxidase blocking in 1% H_2_O_2_ solution in methanol for 15 minutes. Antigen retrieval was performed by autoclaving the sections at 105°C for 10 minutes in Dako Target Retrieval Solution (Dako, Glostrup, Denmark). Serum blocking was performed with antibody 10% normal rabbit serum for 10 minutes. After H_2_O_2_ and serum blocking, the slides were incubated with primary mouse monoclonal anti-CD4 antibody (1:80 dilution; Dako) at room temperature for 20 minutes, and the slides were incubated with primary mouse monoclonal anti-CD8 antibody (1:100 dilution; Dako) at room temperature for 30 minutes. The secondary antibody was biotin-labeled rabbit anti-mouse IgG, IgA, IgM (1:500; Nichirei, Tokyo, Japan). Detection was performed with a DAB kit (Histofine simple stain kit; Nichirei). The sections were counterstained with hematoxylin.

**Figure 8:**
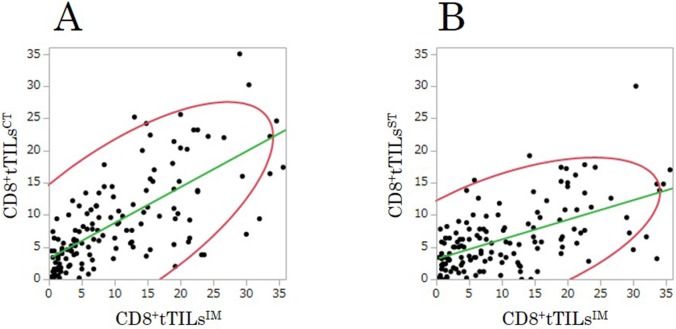
Immune marker expression of tumor-infiltrating lymphocytes **(A)** CD4. **(B)** CD8. Magnification, 400×.

**Figure 9:**
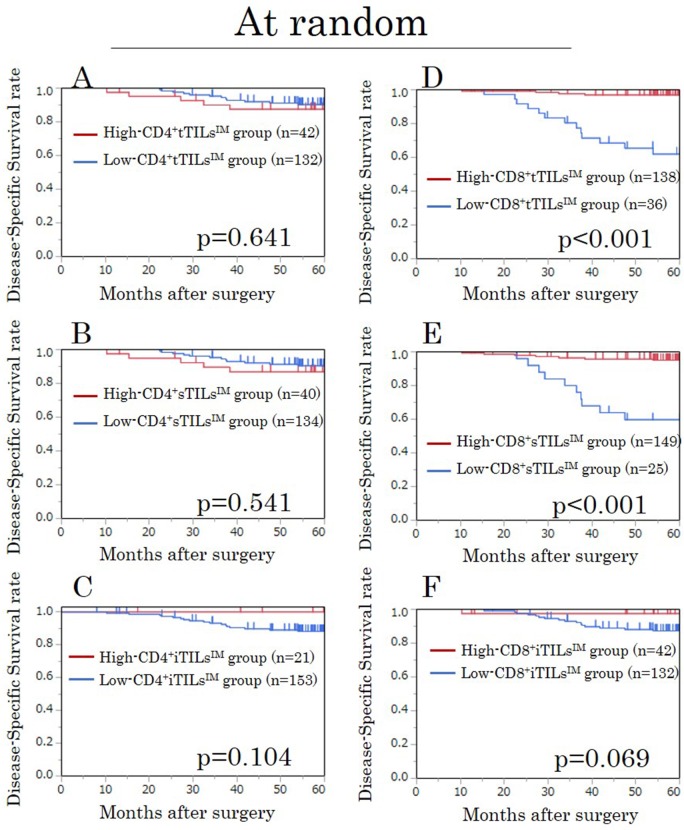
Immunoreactive TILs determined at each intratumoral subsite in low-power fields (invasive margin, the center of the tumor and the surface of the tumor) Hot spots are indicated by an arrow.

**Figure 10:**
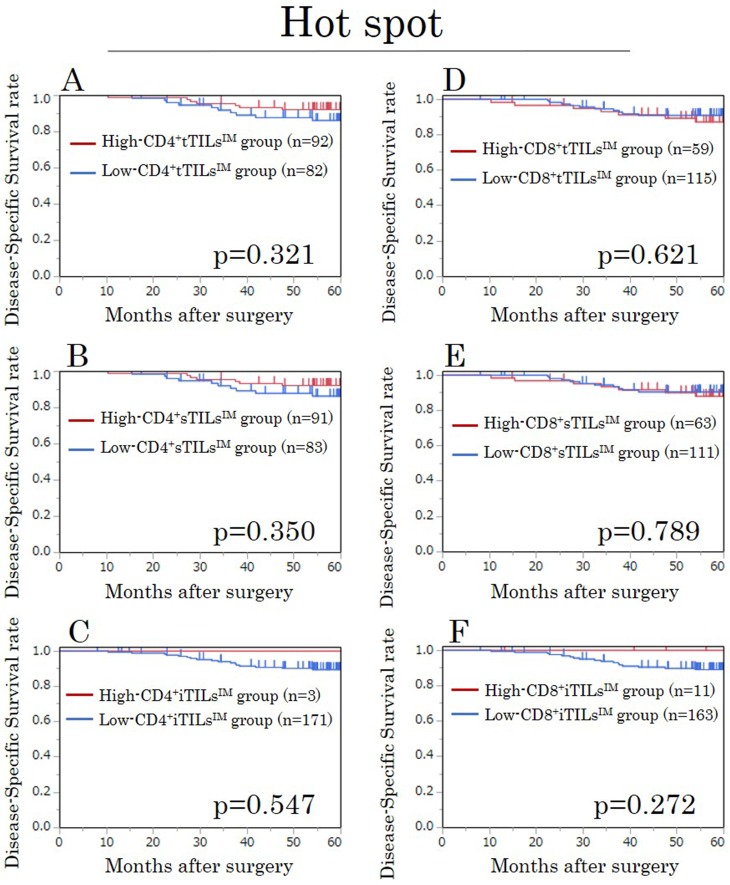
TILs in tumor stroma and intra-tumor cells Arrowhead: iTILs, TILs intra-tumor cells. arrow: sTILs, TILs in tumor stroma.

The immunohistochemical evaluation was carried out by two independent pathologists who were blinded to the clinical information. We examined the average number of TILs in 5 different fields with a light microscope at 400× magnification by the following: 1) subsets of lymphocytes (i.e. CD4^+^(Figure 8A) or CD8^+^(Figure 8B)), 2) selected fields (i.e. at random or focusing on hot spots (Figure 9)), 3) location in low-power field (Figure 9) (i.e. the invasive margin [TILs^IM^] or the center of the tumor [TILs^CT^] or the surface of the tumor [TILs^ST^]), 4) location in high-power field (Figure 10) (i.e. in tumor stroma [sTILs] or intra-tumor cells [iTILs] or total TILs [tTILs: sTILs+iTILs]). We set each median value as the cut-off value for the density of TILs evaluated by each method in the exploratory study (Table 5). In the validation study, we also used the cut-off value used in the exploratory study. We then classified the patients into the high- and low-TILs groups.

**Table 5:** The number of TILs at the invasive margin (/field)

	Exploratory Group		Validation Group	
	At random	Hot spot	At random	Hot spot
CD4 (Median [range])				
tTILs	4.2 (0-36.6)	18.4 (1.2-60.4)	2.2 (0-13.8)	18.8 (4-51.0)
sTILs	4.2 (0-36.6)	18.4 (1.2-60.4)	2.2 (0-13.8)	18.8 (4-51.0)
iTILs	0 (0-0.4)	0 (0-0.2)	0 (0-2.2)	0 (0-4.4)
CD8 (Median [range])				
tTILs	7.6 (0.4-37.8)	39 (9.4-114.2)	12.5 (0.2-34.2)	32.6 (7.0-79.6)
sTILs	5.0 (0-29.0)	37.4 (3.8-114.2)	11.6 (0.2-29.8)	32.6 (7.0-79.6)
iTILs	1.6 (0-16.4)	0 (0-16.8)	0.2 (0-12.8)	0 (0-4.0)

TILs: Tumor-infiltrating lymphocytes, tTILs: total TILs (sTILs+iTILs), sTILs: TILs in tumor stroma, iTILs: TILs intra tumor cells.

### Tissue microarray construction

We constructed a tissue microarray (TMA) in order to evaluate the mismatch repair (MMR) status by immunohistochemical staining. A tissue microarray with one 3.0-mm-diameter punch core per cancer was constructed from formalin-fixed paraffin-embedded tissue blocks of all patients, as previously reported [33]. We ensured that the specific tumor histological type was representatively included in the TMA using Hematoxylin-Eosin-stained TMA sections.

### Immunohistochemistry for mismatch repair status

The effectiveness of an immunohistochemical analysis of the MMR proteins is reportedly similar to that of genotyping for microsatellite instability (MSI) [34]. Therefore, the MSI status was estimated based on the mismatch repair (MMR) status, as previously reported [35]. The MMR status was identified by immunohistochemical staining of MMR proteins (i.e. MLH1, MSH2, MSH6 and PMS2), as previously reported [36].

All 4-μm-thick TMA slides were deparaffined and rehydrated and then subjected to endogenous peroxidase blocking in 1% H_2_O_2_ solution in methanol for 15 minutes. Antigen retrieval was performed by autoclaving the sections at 121°C for 15 minutes in Dako Target Retrieval Solution (Dako). Serum blocking was performed with antibody 10% normal rabbit serum for 10 minutes. After H_2_O_2_ and serum blocking, the slides were incubated in primary antibody for 20 minutes for MLH1 (prediluted product), 20 minutes at a concentration of 1:50 for MSH2 and MSH6, and 30 minutes at a concentration of 1:40 for PMS2 at room temperature (product codes: IS079, M3639, M3646, M3647 [all from Dako]). The secondary antibody was biotin-labeled rabbit anti-mouse IgG, IgA, IgM (1:500; Nichirei). Detection was performed with a DAB kit (Histofine simple stain kit; Nichirei). The sections were counterstained with hematoxylin.

**Figure 11:**
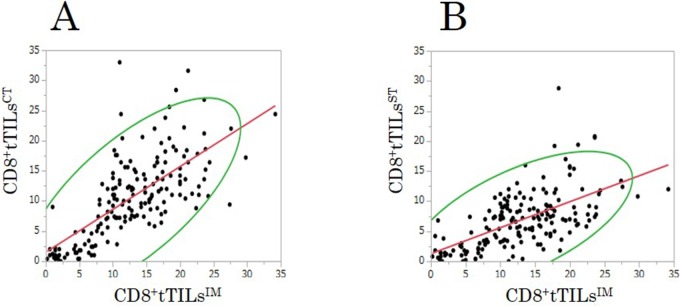
Immunohistochemical staining for mismatch repair proteins MLH1 (**A**. positive, **B**. negative). MSH2 (**C**. positive, **D**. negative). MSH6 (**E**. positive, **F**. negative). PMS2 (**G**. positive, **H**. negative). Magnification, 400×.

The MMR protein expression was evaluated by two pathologists blinded to the clinical outcomes. Normal colon tissue was used as a positive control, and positive staining within intra-tumoral immune cells was used as an internal positive control. The expression was evaluated as MMR-proficient (MMR-P) (tumor cell nuclear expression with positive immune cell expression) (Figure 11A, 11C, 11E, 11G) or MMR-deficient (MMR-D) (absent tumor cell nuclear expression with positive immune cell expression) (Figure 11B, 11D, 11F, 11H). One core was examined per patient for each MMR protein. The tumor was defined as MMR-D when one or more MMR proteins was negatively expressed.

### Statistical analyses

The duration of the survival was calculated using the Kaplan-Meier method. Differences in the survival curves were assessed using the log-rank test. The significance of the correlations between TILs and the clinicopathological characteristics was analyzed using the χ^2^ test and Fisher's exact test. A multivariate analysis was performed according to the Cox proportional hazard model. Associations between the density of TILs^IM^ and the density of TILs^CT^/TILs^ST^ was evaluated using the Pearson's correlation analysis. Associations between the density of TILs in MMR-P tumor and that in MMR-D tumor were analyzed using Wilcoxon's rank sum test. All of the statistical analyses were conducted using JMP^®^ 13.0.0 software program (2016 SAS institute Inc., Cary, NC, USA). P values of <0.05 were considered to indicate statistical significance.

### Ethical considerations

This study conformed to the provisions of the Declaration of Helsinki. All patients were informed of the investigational nature of this study and provided their written informed consent. This retrospective study was approved by the ethics committee of Osaka City University (approved no. 3853).

## Abbreviations

CA19: 9-carbohydrate antigen 19-9
CEA: carcinoembryonic antigen
CI: confidence interval
CRC: Colorectal cancer
DSS: disease-specific survival
HR: hazard ratio
INF: γ-interferon-γ
iTILs: tumor-infiltrating lymphocytes intra-tumor cells
RFS: relapse-free survival
sTILs: tumor-infiltrating lymphocytes in tumor stroma
Th1 cells: T helper 1 cells
TILs: tumor-infiltrating lymphocytes
TILs^CT^: tumor-infiltrating lymphocytes at the center of the tumor
TILs^IM^: tumor-infiltrating lymphocytes at the invasive margin
TILs^ST^: tumor-infiltrating lymphocytes at the surface of the tumor
Treg cells: regulatory T cells
tTILs: total tumor-infiltrating lymphocytes

## References

[1] Howe HL, Wu X, Ries LA, Cokkinides V, Ahmed F, Jemal A, Miller B, Williams M, Ward E, Wingo PA, Ramirez A, Edwards BK (2006). Annual report to the nation on the status of cancer, 1975-2003, featuring cancer among U.S. Hispanic/Latino populations. Cancer.

[2] Rudy DR, Zdon MJ (2000). Update on colorectal cancer. Am Fam Physician.

[3] McArdle CS, Hole DJ (2002). Outcome following surgery for colorectal cancer: analysis by hospital after adjustment for case-mix and deprivation. Br J Cancer.

[4] Mlecnik B, Bindea G, Pages F, Galon J (2011). Tumor immunosurveillance in human cancers. Cancer Metastasis Rev.

[5] Locker GY, Hamilton S, Harris J, Jessup JM, Kemeny N, Macdonald JS, Somerfield MR, Hayes DF, Bast RC (2006). ASCO 2006 update of recommendations for the use of tumor markers in gastrointestinal cancer. J Clin Oncol.

[6] Angell H, Galon J (2013). From the immune contexture to the Immunoscore: the role of prognostic and predictive immune markers in cancer. Curr Opin Immunol.

[7] Fridman WH, Pages F, Sautes-Fridman C, Galon J (2012). The immune contexture in human tumours: impact on clinical outcome. Nat Rev Cancer.

[8] Galon J, Mlecnik B, Bindea G, Angell HK, Berger A, Lagorce C, Lugli A, Zlobec I, Hartmann A, Bifulco C, Nagtegaal ID, Palmqvist R, Masucci GV (2014). Towards the introduction of the ‘Immunoscore’ in the classification of malignant tumours. J Pathol.

[9] Horne ZD, Jack R, Gray ZT, Siegfried JM, Wilson DO, Yousem SA, Nason KS, Landreneau RJ, Luketich JD, Schuchert MJ (2011). Increased levels of tumor-infiltrating lymphocytes are associated with improved recurrence-free survival in stage 1A non-small-cell lung cancer. J Surg Res.

[10] Pages F, Kirilovsky A, Mlecnik B, Asslaber M, Tosolini M, Bindea G, Lagorce C, Wind P, Marliot F, Bruneval P, Zatloukal K, Trajanoski Z, Berger A (2009). In situ cytotoxic and memory T cells predict outcome in patients with early-stage colorectal cancer. J Clin Oncol.

[11] Pelletier MP, Edwardes MD, Michel RP, Halwani F, Morin JE (2001). Prognostic markers in resectable non-small cell lung cancer: a multivariate analysis. Can J Surg.

[12] Schumacher K, Haensch W, Roefzaad C, Schlag PM (2001). Prognostic significance of activated CD8(+) T cell infiltrations within esophageal carcinomas. Cancer Res.

[13] Wahlin BE, Sander B, Christensson B, Kimby E (2007). CD8+ T-cell content in diagnostic lymph nodes measured by flow cytometry is a predictor of survival in follicular lymphoma. Clin Cancer Res.

[14] Galon J, Pages F, Marincola FM, Thurin M, Trinchieri G, Fox BA, Gajewski TF, Ascierto PA (2012). The immune score as a new possible approach for the classification of cancer. J Transl Med.

[15] Kim Y, Bae JM, Li G, Cho NY, Kang GH (2015). Image analyzer-based assessment of tumor-infiltrating T cell subsets and their prognostic values in colorectal carcinomas. PLoS One.

[16] Nguyen N, Bellile E, Thomas D, McHugh J, Rozek L, Virani S, Peterson L, Carey TE, Walline H, Moyer J, Spector M, Perim D, Prince M, Head Neck SPORE Program Investigators (2016). Tumor infiltrating lymphocytes and survival in patients with head and neck squamous cell carcinoma. Head Neck.

[17] Noble F, Mellows T, McCormick Matthews LH, Bateman AC, Harris S, Underwood TJ, Byrne JP, Bailey IS, Sharland DM, Kelly JJ, Primrose JN, Sahota SS, Bateman AR (2016). Tumour infiltrating lymphocytes correlate with improved survival in patients with oesophageal adenocarcinoma. Cancer Immunol Immunother.

[18] Xie Y, Akpinarli A, Maris C, Hipkiss EL, Lane M, Kwon EK, Muranski P, Restifo NP, Antony PA (2010). Naive tumor-specific CD4(+) T cells differentiated in vivo eradicate established melanoma. J Exp Med.

[19] Galon J, Angell HK, Bedognetti D, Marincola FM (2013). The continuum of cancer immunosurveillance: prognostic, predictive, and mechanistic signatures. Immunity.

[20] Obeid JM, Wages NA, Hu Y, Deacon DH, Slingluff CL (2017). Heterogeneity of CD8+ tumor-infiltrating lymphocytes in non-small-cell lung cancer: impact on patient prognostic assessments and comparison of quantification by different sampling strategies. Cancer Immunol Immunother.

[21] Mahmoud SM, Paish EC, Powe DG, Macmillan RD, Grainge MJ, Lee AH, Ellis IO, Green AR (2011). Tumor-infiltrating CD8+ lymphocytes predict clinical outcome in breast cancer. J Clin Oncol.

[22] Ling A, Edin S, Wikberg ML, Oberg A, Palmqvist R (2014). The intratumoural subsite and relation of CD8(+) and FOXP3(+) T lymphocytes in colorectal cancer provide important prognostic clues. Br J Cancer.

[23] Richards CH, Roxburgh CS, Powell AG, Foulis AK, Horgan PG, McMillan DC (2014). The clinical utility of the local inflammatory response in colorectal cancer. Eur J Cancer.

[24] Galon J, Pages F, Marincola FM, Angell HK, Thurin M, Lugli A, Zlobec I, Berger A, Bifulco C, Botti G, Tatangelo F, Britten CM, Kreiter S (2012). Cancer classification using the Immunoscore: a worldwide task force. J Transl Med.

[25] Galon J, Costes A, Sanchez-Cabo F, Kirilovsky A, Mlecnik B, Lagorce-Pages C, Tosolini M, Camus M, Berger A, Wind P, Zinzindohoue F, Bruneval P, Cugnenc PH (2006). Type, density, and location of immune cells within human colorectal tumors predict clinical outcome. Science.

[26] Ishikawa T, Fujita T, Suzuki Y, Okabe S, Yuasa Y, Iwai T, Kawakami Y (2003). Tumor-specific immunological recognition of frameshift-mutated peptides in colon cancer with microsatellite instability. Cancer Res.

[27] Phillips SM, Banerjea A, Feakins R, Li SR, Bustin SA, Dorudi S (2004). Tumour-infiltrating lymphocytes in colorectal cancer with microsatellite instability are activated and cytotoxic. Br J Surg.

[28] Gavin PG, Colangelo LH, Fumagalli D, Tanaka N, Remillard MY, Yothers G, Kim C, Taniyama Y, Kim SI, Choi HJ, Blackmon NL, Lipchik C, Petrelli NJ (2012). Mutation profiling and microsatellite instability in stage II and III colon cancer: an assessment of their prognostic and oxaliplatin predictive value. Clin Cancer Res.

[29] Shinto E, Hase K, Hashiguchi Y, Sekizawa A, Ueno H, Shikina A, Kajiwara Y, Kobayashi H, Ishiguro M, Yamamoto J (2014). CD8+ and FOXP3+ tumor-infiltrating T cells before and after chemoradiotherapy for rectal cancer. Ann Surg Oncol.

[30] Teng F, Meng X, Kong L, Mu D, Zhu H, Liu S, Zhang J, Yu J (2015). Tumor-infiltrating lymphocytes, forkhead box P3, programmed death ligand-1, and cytotoxic T lymphocyte-associated antigen-4 expressions before and after neoadjuvant chemoradiation in rectal cancer. Transl Res.

[31] Teng F, Mu D, Meng X, Kong L, Zhu H, Liu S, Zhang J, Yu J (2015). Tumor infiltrating lymphocytes (TILs) before and after neoadjuvant chemoradiotherapy and its clinical utility for rectal cancer. Am J Cancer Res.

[32] Salgado R, Denkert C, Demaria S, Sirtaine N, Klauschen F, Pruneri G, Wienert S, Van den Eynden G, Baehner FL, Penault-Llorca F, Perez EA, Thompson EA, Symmans WF, International TILs Working Group 2014 (2015). The evaluation of tumor-infiltrating lymphocytes (TILs) in breast cancer: recommendations by an International TILs Working Group 2014. Ann Oncol.

[33] Milanes-Yearsley M, Hammond ME, Pajak TF, Cooper JS, Chang C, Griffin T, Nelson D, Laramore G, Pilepich M (2002). Tissue micro-array: a cost and time-effective method for correlative studies by regional and national cancer study groups. Mod Pathol.

[34] Hampel H, Frankel WL, Martin E, Arnold M, Khanduja K, Kuebler P, Nakagawa H, Sotamaa K, Prior TW, Westman J, Panescu J, Fix D, Lockman J (2005). Screening for the Lynch syndrome (hereditary nonpolyposis colorectal cancer). N Engl J Med.

[35] Dudley JC, Lin MT, Le DT, Eshleman JR (2016). Microsatellite Instability as a Biomarker for PD-1 Blockade. Clin Cancer Res.

[36] Hu J, Yan WY, Xie L, Cheng L, Yang M, Li L, Shi J, Liu BR, Qian XP (2016). Coexistence of MSI with KRAS mutation is associated with worse prognosis in colorectal cancer. Medicine (Baltimore).

